# Supplementary material for: CSN and CAVA: variant annotation tools for rapid, robust next-generation sequencing analysis in the clinical setting
Source: Genome Med. 2015 Jul 28;7(1):76. doi: 10.1186/s13073-015-0195-6 (PMC4551696; doi:10.1186/s13073-015-0195-6)
Supplement: Additional file 1: — Clinical sequencing nomenclature (CSN) description. (DOCX 40 kb) [file 13073_2015_195_MOESM1_ESM.docx]

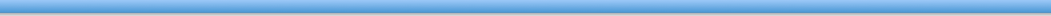


**THE CLINICAL SEQUENCING NOMENCLATURE (CSN) v1.0**

**Background**

A fixed, standardized, versioned nomenclature for reporting clinical sequence data, identical for all mutation detection platforms and readily interchangeable with historic data is of vital importance. It allows integration of sequencing data from multiple sources and facilitates more accurate clinical interpretation of genomic information. The Clinical Sequencing Nomenclature (CSN) aims to achieve this. It follows the principles of the existing HGVS nomenclature [1], with minor amendments to ensure compatibility and integration of historical clinical sequence data, whilst also allowing high-volume automated output from NGS platforms.

**The aims of CSN**

The aims of CSN are

- To provide a fixed, standardized system in which each variant has a single notation
- To use a logical terminology understandable to non-experts
- To provide a nomenclature that allows easy visual discrimination between the major classes of variant in clinical genomics

**Description of CSN**

The CSN is described in detail below. General points:

- CSN provides nomenclature for three basic types of variant, defined at the nucleotide level
  - Substitution: a change of one base to another
  - Indel: insertion or deletion of one or more bases
  - Complex: a change involving consecutive base other than simple loss or gain of sequence.
- Other types of variation exist (e.g. inversions, conversions, or translocations) but are not currently encompassed by the CSN.
- All CSN descriptions represent a variant in a single allele.
- All CSN variants are reported with a single descriptor. Nucleotide and amino acid level descriptions are joined with an underscore.

Use of the CSN requires the following:

- Reference sequence transcript
- Variant sequence to be annotated

**The reference sequence transcript**

The reference sequence transcript is the nucleotide (DNA) and amino acid (protein) sequence against which variation in the sequence being annotated is described.

The nucleotide sequence defines the amino acid sequence according to the genetic triplet code: three nucleotides (termed a ‘codon’) code for one amino acid [2].

The transcript includes:

- Exons – regions of sequence which are translated into the protein. The protein code starts with a methionine and ends with a stop codon.
- Introns – regions of sequence between exons that are ‘spliced’ out and not included in the protein
- UTR – regions that are transcribed but not translated – the region before the protein starts is called the 5’ UTR and the region after the protein ends is called the 3’ UTR.

**Reference sequence position numbering**

- The sequence is numbered from the 5’ to 3’ direction of the transcript.
- Nucleotide (DNA) sequence level positions are prefaced with “c.”
- Amino acid (protein) sequence level positions are prefaced with “p.”
- “c.1” is always the first protein-coding base: “A” of the initiating methionine.
- “p.1” is always the initiating methionine.
- Positions within the intron and UTR are numbered in relation to the nearest exon. Positions 5’ of the nearest exon are denoted with “-”. Positions 3’ of the nearest exon are denoted with “+”. Positions 3’ of the translation termination codon are denoted with “*”.
- A base that occupies the central position within an intron comprised of an odd number of bases, i.e. equidistant from neighboring exons, is assigned “+”.

Examples:

| c.10T | The tenth protein-coding base. |
| --- | --- |
| c.-10T | The tenth base position in the sequence situated 5’ of the translation initiation codon. |
| c.*10T | The tenth base position in the sequence situated 3’ of the translation termination codon. |
| c.100+10T | Position c.100 is the final coding base of the exon.  The base indicated is the tenth intronic base, situated 3’ from the splice donor site. |
| c.101-10G | Position c.101 is the first coding base of the exon.  The base indicated is the tenth intronic base, situated 5’ to the splice acceptor site. |

**Variant description**

- The CSN uses the three letter code to describe amino acids [2] with the exception of ‘X’ which is used to describe any stop codon.
- A single descriptor with the nucleotide and amino acid (if appropriate) level changes, linked with an underscore is given.
- Variants that do not change the amino acid (synonymous) are described at the amino acid level with “p.=”
- Variants that change the amino acid (nonsynonymous, also called missense) are described at the amino acid level with the reference amino acid(s), the position, and the variant amino acid(s).
- Stop-gain variants (also called nonsense) are designated at the amino acid level with ‘X’.
- Variants which change the initiating methionine codon are described at the amino acid level as “p.Met1?”
- Variants which result in loss of the termination codon (stop-loss variants) are described at the amino acid level as “p.extX” followed by either the length of the predicted amino acid extension when a subsequent stop codon exists within the continued reading frame, or “?” when no subsequent stop codon exists within the 3’ UTR sequence along the continued reading frame.
- Only nucleotide level information is provided for variants in UTR or intronic sequence.
- Indel variants and complex variants which alter the protein sequence length can either shift the reading frame (frameshifting) or leave the reading frame unaltered (inframe).
- Only a nucleotide level description is given for frameshifting variants. Most lead to nmRNA decay and removal of the transcript, rather than a truncated protein [3]
- Indels and complex variants with multiple possible representations according to the reference sequence are described in the CSN at the most 3’ position in the coding transcript.

**Substitution variants**

Substitution variants are those in which one base is ‘substituted’ for another base. They are described at the nucleotide level with the position, followed by the reference base(s), “>”, and the variant base(s) and at the amino acid level according to the impact.

Examples:

| c.99A>C_p.= | C is substituted for base 99 (A). There is no change to the amino acid (synonymous variant). |
| --- | --- |
| c.99A>C_p.Gln33His | C is substituted for base 99 (A), resulting in histidine replacing glutamine (nonsynonymous variant). |
| c.78-3G>T | T is substituted for the base positioned three bases 5’ of the first base (78) of the nearest exon. This is an intronic variant. |
| c.97A>T_p.Lys33X | T is substituted for base 97 (A), resulting in a stop codon replacing lysine (stop-gain variant). |
| c.999A>C_p.extX? | C is substituted for base 999 (A), resulting in a change in the termination codon and no new stop codon exists within the 3’ UTR sequence along the continued reading frame (stop-loss variant). |
| c.999A>C_p.extX12 | C is substituted for base 999 (A), resulting in a change in the termination codon with a predicted extension by 12 amino acids where the next stop codon exists within the continued reading frame (stop-loss variant). |
| c.3G>A_p.Met? | A is substituted for base 3 (G), resulting in a change in the initiating methionine codon. |

**Indel variants**

Indel variants can be further classified into three categories: deletions, insertions and duplications. Each category is described below.

**Deletions**

Deletions are described at the nucleotide level with the position of the deleted bases followed by “del” and either the deleted bases (deletions of 1-4 bases) or the number of deleted bases (deletions of 5 or more bases).

When more than one base is deleted, the positions of the deleted bases are described as the first and last deleted positions separated by an underscore.

Examples:

| c.100delG | The single base 100 is deleted causing a frameshift. |
| --- | --- |
| c.121+5delG | The base positioned five bases 3’ of the final base (121) of the nearest exon is deleted. This is an intronic variant. |
| c.100_102delTTT_p.Phe34del | Three bases are deleted, resulting in an inframe deletion of phenylalanine. |
| c.100_108del9_p.Phe34_Met36del | Nine bases are deleted, resulting in an inframe deletion of three amino acids. |
| c.107_109delTGC_p.Met36_Pro37delinsThr | Three bases are deleted, resulting in threonine replacing methionine and proline. |

**Insertions**

Insertions are described at the nucleotide level with the position of the insertion followed by “ins” and the inserted bases.

The position of the insertion is described by the two flanking bases of the reference sequence separated by an underscore, thus the numbers presented are always consecutive.

Examples:

| c.76_77insT | T is inserted between bases 76 and 77 causing a frameshift. |
| --- | --- |
| c.77+4_77+5insAA | AA is inserted between the fourth and fifth bases 3’ of the final base (77) of the nearest exon. This is an intronic variant. |
| c.101_102insCTG_p.Phe34_Thr35insCys | Three bases are inserted between bases 101 and 102, resulting in an inframe insertion of cysteine. |
| c.103_104insGGT_p.Thr35delinsArgSer | Three bases are inserted between bases 103 and 104, resulting in arginine and serine replacing threonine. |

**Duplications:**

Duplications are a specific category of insertion where the inserted base sequence is a duplication of the immediately preceding reference sequence.

- Duplications can comprise any number of bases, but the category does not include an inserted sequence that duplicates a preceding reference sequence motif more than once.
- Duplications are described at the nucleotide level with the positions of the duplicated sequence followed by “dup” and either the duplicated bases (duplications of 1-4 bases) or the number of duplicated bases (duplications of 5 or more bases).
- The positions of the duplicated sequence are defined by reference to the duplicated reference positions, not the point of insertion.
- When more than one base is duplicated, the positions of the duplicated bases are described as the first and last duplicated positions separated by an underscore.

Examples:

| c.5dupT | The single base 5 is duplicated causing a frameshift. |
| --- | --- |
| c.5_9dup5 | The five bases 5-9 are duplicated causing a frameshift. |
| c.7_12dup6_p.His3_Pro4dup | The six bases 7-12 are duplicated, resulting in the duplication of histidine and proline. |

**Complex variants**

Complex variants are those which involve consecutive bases but are not simple deletions or insertions. They are described at the nucleotide level with the positions of the deleted bases followed by “delins” and the inserted nucleotides.

- Variants involving consecutive bases are described as a single complex variant rather than separate multiple events such as a deletion followed by a substitution.
- Complex variants may or may not result in a change in sequence length.
- Complex variants can be frameshifting or inframe variants (complex indel) or result in no change in sequence length (complex substitution).
- The positions of the deleted bases are described by the range of bases that are lost and being replaced.
- When more than one base is deleted, the positions of the deleted bases are described as the first and last deleted positions separated by an underscore.

Examples:

| c.2854_2855delinsAT_p.Ala952Met | The two bases 2854-2855 are replaced with AT, resulting in methionine replacing alanine (complex substitution). |
| --- | --- |
| c.112_116delinsTG | The five bases 112-116 are replaced with TG resulting in a frameshift (complex indel). |
| c.100_102delinsGTTAAG_p.Ser34delinsValLys | The three bases 100-102 are replaced with six bases, resulting in valine and lysine replacing serine (complex indel). |

**References**

1. den Dunnen, J.T. and S.E. Antonarakis, *Nomenclature for the description of human sequence variations.* Hum Genet, 2001. **109**(1): p. 121-4.

2. Codons and amino acids (Nomenclature for the description of sequence variants: codons and amino acids) http://www.hgvs.org/mutnomen/codon.html

3. Chang, Y. F; Imam, J. S and Wilkinson, M. F. (2007). *The nonsense-mediated decay RNA surveillance pathway*. Annual Review of Biochemistry, 2007 **76**: 51–74.
