# Supplementary material for: CSN and CAVA: variant annotation tools for rapid, robust next-generation sequencing analysis in the clinical setting
Source: Genome Med. 2015 Jul 28;7(1):76. doi: 10.1186/s13073-015-0195-6 (PMC4551696; doi:10.1186/s13073-015-0195-6)
Supplement: Additional file 2: — Clinical Annotation of Variants (CAVA) description. (DOCX 88 kb) [file 13073_2015_195_MOESM2_ESM.docx]

**CAVA v1.0.0**

**documentation**

**CONTENTS**

**1** INTRODUCTION --------------*2*

**2** INSTALLATION --------------*2*

**3** RUNNING CAVA --------------*2*

**4** CONFIGURATION FILE --------*3*

**5** INPUT FILE ----------------*5*

**6** REFERENCE GENOME ----------*5*

**7** TRANSCRIPT DATABASE -------*5*

**8** DBSNP DATABASE ------------*6*

**9** VARIANT ANNOTATION --------*6*

**10** THE CLASS ONTOLOGY ------*10*

**11** OUTPUT ------------------*12*

**12** VARIANT CALL FILTERING --*15*

**13** THE DBPREP TOOL ---------*17*

**14** DEFAULT TRANSCRIPT

AND DBSNP DATABASE ------*18*

**15** RUN TIMES ---------------*19*

**16** LOG FILE ----------------*19*

**17** EXAMPLES ----------------*20*

**18** CONTACT -----------------*26*


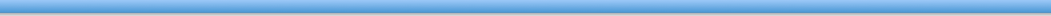


**1 INTRODUCTION**

CAVA (Clinical Annotation of Variants) is a lightweight, flexible, fast, easy-to-use variant annotation tool specializing in gene- (transcript-) level variant call annotation. This detailed documentation describes all features of CAVA and its accompanying database preparation tool, dbprep. After first introducing its user interface and discussing its functionalities, some examples are presented in the second part of this documentation.


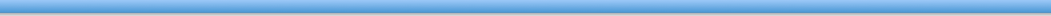


**2 INSTALLATION**

CAVA can be downloaded from *www.well.ox.ac.uk/cava*

After unpacking the tar.gz file, CAVA is ready for variant annotation.

The tar.gz file also contains the database preparation tool, dbprep, and a template configuration file.

Additional files (i.e. default whole exome transcript and dbSNP databases) can be downloaded from the same webpage.

**2.1 Dependencies**

CAVA v1.0.0 requires Python (version 2.6 or later) and the Pysam module (version 0.7.7 or later) (together with its dependency; cython) to be installed*:* [*https://github.com/pysam-developers/pysam*](https://github.com/pysam-developers/pysam)


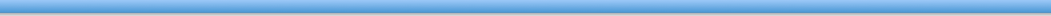


**3 RUNNING CAVA**

Once downloaded with dependencies correctly installed (see above), CAVA can be run with the following simple command:

**python path/to/cava/cava.py -c config.txt -i input.vcf -o output**

The program requires three command line arguments: the name of the configuration file (-c), the name of the input file (-i) and the prefix of the output file name (-o).

The configuration file should contain all user-specified settings (see below). The input file should contain the set of variant calls to be annotated. This file can be in standard VCF format or a TXT file (see below for possible input formats). Finally, the output prefix is used to create the name of the output file and the log file.

Optionally, by using option -s, the annotated variant set will be written to the standard output (stdout) rather than to an output file (however, a log file will still be created, if required).

Alternatively, help information on the command line arguments can be requested using the -h argument:

**python path/to/cava/cava.py -h**


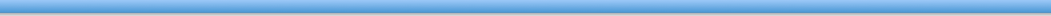


**4 CONFIGURATION FILE**

As mentioned above, the configuration file should contain all user-specified options. Here we describe the list of possible option flags (see more detailed discussions in next sections). Example configuration files are also given in Section 17 (and in the cava-v1.1.0.tar.gz file).

Each option set in the configuration file should be given in the format of

@flagname = value

CAVA understands the following option flags (some are required and some are optional with the default values shown below):

**@inputformat**

Input file format. (Optional. Possible values: VCF or TXT. Default value: VCF)

**@outputformat**

Output file format. (Optional. Possible values: VCF or CSV. Default value: VCF)

**@reference**

Name of reference genome file. (Required.)

**@ensembl**

Name of Ensembl transcript database file. (Optional. If not given, no transcript-based annotation will be performed.)

**@dbsnp**

Name of dbSNP database file. (Optional. If not given, no SNP-based annotation will be performed.)

**Note: using at least one of @ensembl or @dbsnp options is required!**

**@nonannot**

Boolean flag specifying if variants which received neither transcript nor dbSNP annotations are to be included in the output. (Optional. Possible values: TRUE or FALSE. Default value: TRUE)

**@filter**

Boolean flag specifying if only records with PASS filter value are included in the output. (Optional. Possible values: TRUE or FALSE. Default value: FALSE)

**@type**

Types of variants to be annotated. (Optional. Possible values: ALL, SUBSTITUTION, INDEL, INSERTION, DELETION or COMPLEX. Default value: ALL)

**@target**

Name of compressed BED file specifying genomic regions variant annotation is restricted to. (Optional.)

**@genelist**

Name of file providing a list of the gene identifiers variant annotation is restricted to. Gene identifiers are to be given on separate lines in the file. (Optional.)

**@transcriptlist**

Name of file providing a list of the transcript identifiers variant annotation is restricted to. Transcript identifiers are to be given on separate lines in the file. (Optional.)

**@snplist**

Name of file providing a list of the dbSNP identifiers variant annotation is restricted to. dbSNP identifiers are to be given on separate lines in the file. (Optional.)

**@logfile**

Boolean flag specifying if log file is written. (Optional. Possible values: TRUE or FALSE. Default value: FALSE)

**@ontology**

Which ontology is used for reporting functional class assignment. (Optional. Possible values: CLASS, SO or BOTH. Default value: BOTH)

**@impactdef**

Definition of variant impact levels (reported by the IMPACT annotation flag)

**@givealt**

Boolean flag specifying if alternative most 5’ and CLASS/SO annotations are to be outputted. (Optional. Possible values: TRUE or FALSE. Default value: TRUE)

**@ssrange**

Number of bases into the intron defined as the splice site region (as used in the CLASS flag). (Optional. Possible values: integer >= 6. Default value: 8)


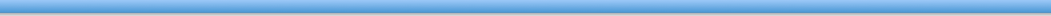


**5 INPUT FILE**

The input file (defined by command line argument -i) contains all variant calls to be annotated. It may follow two formats, VCF or TXT (specified in the configuration file by option @inputformat).

**5.1 Input in VCF format**

If VCF format is used, the input file should follow the standard [VCF 4.1 specification](http://www.1000genomes.org/wiki/Analysis/Variant%20Call%20Format/vcf-variant-call-format-version-41). A single VCF record can describe a multiallelic variant which will be considered by CAVA as multiple different variant calls. VCF records may also include genotype call information.

**5.2 Input in TXT format**

If TXT format is used, the input file must be written in the following tab-delimited 5-column format describing the variant ID, chromosome name, genomic position, reference allele and comma-separated alternative alleles:

#ID CHROM POS REF ALT

1 13 1324552 G A,TC

…

Note that this format does not contain information about the filter value, therefore filter=PASS will be set for each variant.


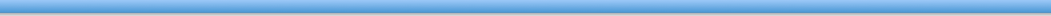


**6 REFERENCE GENOME**

In order to annotate variants, CAVA requires a reference genome sequence. The reference genome (specified in the configuration file by option @reference) must be a FASTA file indexed with *samtools faidx*. Both the FASTA file and the index file (e.g. *hg19.fa* and *hg19.fa.fai*) should be available in the same directory.

Chromosome names in the FASTA file can either be of the format '14' or 'chr14'.


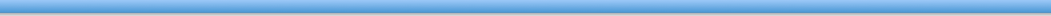


**7 TRANSCRIPT DATABASE**

To perform Ensembl transcript-based annotation, CAVA requires a local transcript database that contains all relevant information about each transcript (strandedness, genomic positions of exons etc.). The transcript database file needs to have a special format, compressed with bgzip and indexed with Tabix. A simple tool (dbprep) is provided to generate the compressed and Tabix-indexed transcript database file based on an arbitrary list of transcript IDs (see Section 13 for a detailed description of the dbprep tool). Alternatively, one can use the readily available whole exome transcript database (see Section 14) which can be downloaded from *www.well.ox.ac.uk/cava*.

The name of the transcript database file is specified in the configuration file by the option @ensembl. Note that at least one of @ensembl or @dbsnp must be given.


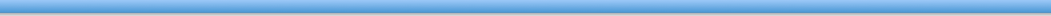


**8 DBSNP DATABASE**

In order to perform SNP annotation, CAVA requires a local dbSNP database that contains information about each SNP (i.e. its identifier and genomic position). The dbSNP database file needs to have a special format compressed with bgzip and indexed with Tabix. As with the case of transcript database discussed above, the simple tool dbprep can be used to generate the compressed and Tabix-indexed dbSNP database file based on an arbitrary list of dbSNP IDs (see Section 13 for a detailed description of the dbprep tool). Alternatively, one can use the readily available dbSNP database files (see Section 14), which can be downloaded from *www.well.ox.ac.uk/cava*.

The name of the dbSNP database file is specified in the configuration file by option @dbsnp. Note that at least one of @ensembl or @dbsnp must be given.


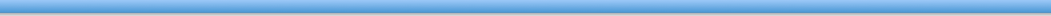


**9 VARIANT ANNOTATION**

CAVA outputs information about variant calls in various annotation flags. Two types of variant annotation are supported: transcript-based and SNP-based. In transcript-based annotation, variant calls are annotated based on the information stored in the local Ensembl transcript database file. In SNP-based annotation, variants are annotated based on the local dbSNP database file.

This section discusses all components of variant annotation. Section 11 describes the exact way these components are written into different output formats.

One annotation flag which is always outputted (regardless of performing transcript-based or SNP-based annotation) is 'TYPE' which refers to the type of variant call (e.g. TYPE=SUBSTITUTION). This annotation flag has four possible values: SUBSTITUTION, INSERTION, DELETION, and COMPLEX, referring to base substitutions, insertions, deletions, and complex variants, respectively.

**9.1 Transcript-based annotation**

If option flag @ensembl is set in the configuration file, CAVA will search the transcript database for each variant call to find Ensembl transcripts that overlap with the variant. Note that CAVA allows overlapping transcripts and a variant may overlap with multiple Ensembl transcripts (see Section 11.1.2).

**9.1.1 The ENST, GENE and TRINFO annotation flags**

Variant calls overlapping with at least one transcript are annotated with the Ensembl transcript identifiers in the annotation flag 'ENST' (e.g. ENST=ENST00000380152).

Transcript-overlapping variants are also annotated with the corresponding gene names (i.e. HGNC symbols) in the annotation flag 'GENE' (e.g. GENE=BRCA2). Basic information about the transcripts (strandedness, length of transcript, number of exons and length of coding DNA, respectively) are added in the 'TRINFO' annotation flag (e.g. TRINFO=+/319.1kb/6/5.3kb). For example, a TRINFO annotation flag value of ‘+/319.1kb/6/5.3kb’ means that the transcript is forward stranded (+), with a total sequence length of 319.1 kb, including 6 exons which together cover 5.3 kb of coding region.

**9.1.2 The LOC annotation flag**

The location of the variant within the transcript is added in the annotation flag 'LOC' (e.g. LOC=Ex8). As exemplified below, the LOC flag may refer to an exon, an intron, a UTR3 or a UTR5 region.

Alternatively, if a variant overlaps the boundary between two different regions (e.g. exon and intron), this is referred to as 'LOC=L1-L2', where L1 and L2 are the locations of the two endpoints of the variant.

**Examples:**

Variant within exon 12: LOC=Ex12

Variant within the intron separating exons 5 and 6: LOC=In5/6

Variant within 5' untranslated region: LOC=UTR5

Variant within 3' untranslated region: LOC=UTR3

Variant overlapping the 3' boundary of exon 7: LOC=Ex7-In7/8

Variant overlapping exon 28 and the 3' untranslated region: LOC=Ex28-UTR3

Note that some Ensembl transcripts contain artificial introns of very short length (1,2,4 or 5 bp) called ‘frameshift introns’, added by the Ensembl genebuild. Since these may indicate errors in either the reference genome assembly or in the aligned cDNA, variants overlapping frameshift introns may actually be exonic or not real. CAVA thus flags variants affecting these introns, reporting this information in the LOC annotation flag, e.g. LOC=fsIn8/9 is a reference to frameshift intron 8/9.

**9.1.3 The CSN, CLASS, SO, IMPACT and ALTFLAG annotation flags**

**The CSN flag:**

The Clinical Sequencing Nomenclature (CSN) v1.0 is used for clinical variant annotation. It is based on the Human Genome Variation Society (HGVS) with minor amendments to allow high-volume automated outputs from NGS pipelines. The CSN is described in detail in Supplementary Appendix 1.

The CSN annotation of a variant affecting a transcript is outputted in the annotation flag 'CSN' (e.g. CSN=c.421A>G_p.Thr141Ala). For indels, CSN is created based on the correctly aligned (strand-aware) representation of the variant. Note that the CSN annotation is given both on DNA (c.) and protein (p.) level if appropriate (otherwise only c. is given).

**The CLASS and SO flags:**

Variants are also classified according to a simple ontology and the class of variant is outputted in the annotation flag 'CLASS' (e.g. CLASS=SY). See Section 10 for detailed description of the CLASS ontology and the definition of different classes. Alternatively, one can output the Sequence Ontology (SO) annotation instead of the CLASS ontology or both ontologies can be reported. One can use the @ontology flag in the configuration file to control which ontologies are outputted. The @ontology flag has three possible values: CLASS, SO or BOTH, specifying that the CLASS ontology, the SO ontology or both are reported, respectively. The default value is BOTH, thus by default both CLASS and SO are outputted. For the description of the SO classification, please refer to the Sequence Ontology website: <http://www.sequenceontology.org/>. For a comparison of the CLASS and SO ontologies, see the table in Section 10.

**The IMPACT flag:**

CAVA can also stratify variants into groups of likely similar impact based on their CLASS annotations. This information is reported in the IMPACT annotation flag. The value of the IMPACT flag is an integer indicating decreasing level of impacts, with the following default corresponding CLASS values:

| **IMPACT** | **CLASS** |
| --- | --- |
| 1 | ESS, FS, SG |
| 2 | NSY, SS5, IF, IM, SL, EE |
| 3 | SY, SS, INT, 5PU, 3PU |

Furthermore, one can define a custom mapping from CLASS terms to IMPACT values by using the @impactdef option flag in the configuration file. Different impact levels are separated by | and a comma-separated list of CLASS terms must be given for each level. For example, the following line defines the default mapping described above:

@impactdef=ESS,FS,SG | NSY, SS5,IF,IM,SL,EE | SY,SS,3PU,5PU,INT

Note that the IMPACT annotation flag is always outputted unless “@impactdef=.” is set in the configuration file. The IMPACT flag is reported even if the CLASS flag is not outputted (i.e. @ontology=SO).

**The ALTFLAG flag:**

Some indels have alternative representations which may even change their CLASS or SO annotations. CAVA recognizes variants with alternative annotations and outputs this information in the annotation flag 'ALTFLAG' (e.g. ALTFLAG=None). If, for example, only the CLASS ontology is reported, the ALTFLAG flag has three possible values: 'None', 'AnnNotClass' and 'AnnAndClass'. If the variant has the same annotation regardless of its left or right alignment, ALTFLAG=None is given. If an indel has an alternative annotation but the same CLASS annotation for both representations, ALTFLAG=AnnNotClass is given. Finally, if the indel has different CLASS annotations depending on its annotation, ALTFLAG=AnnAndClass is outputted, referring to the fact that the different representations may be interpreted as having different functional consequences.

Similarly, when only the SO ontology is reported, the ALTFLAG annotation flag has the following three possible values: 'None', 'AnnNotSO' and 'AnnAndSO'.

If both CLASS and SO ontologies are reported, the possible values of ALTFLAG are: ‘None’, ‘AnnNotClassNotSO’, ‘AnnAndClassNotSO’, ‘AnnAndSONotClass’, ‘AnnAndClassAndSO’, indicating whether the different indel representations would result in the same annotation and same CLASS and/or SO annotations.

**9.1.4 Outputting alternative annotations: ALTANN and ALTCLASS/ALTSO**

If the @givealt option flag is set to TRUE in the configuration file, CAVA will output the most 5’ alternative sequence and CLASS and/or SO annotations instead of just indicating that alternative annotations exist. If the @givealt option is switched on, additional annotation flags (ALTANN and ALTCLASS and/or ALTSO) are given for each variant, providing these alternative sequence and CLASS and/or SO annotations (if any), respectively. If relevant, the most 3’ effect on the protein is outputted in the ALTANN annotation. Note, that if @givealt is used, the ALTFLAG annotation flag described above is not reported.

**9.1.5 Variants located outside transcripts**

Note that the transcript-based annotations described above are only given if both endpoints of the variant are located within the transcript. If there is only partial overlap between the variant and the transcript (e.g. the starting position of a deletion lies within the transcript but the ending position lies outside), the partial overlap is reported in the following way:

The ENST, GENE and TRINFO flags describing the transcript are given as above, however LOC=OUT is reported and empty CSN, CLASS, SO, ALTFLAG, ALTANN, ALTCLASS and ALTSO flag values are outputted (e.g. CSN=.).

If no transcripts are found in the transcript database with which the variant call overlaps, all transcript-specific annotation flags (ENST, GENE, TRINFO, LOC, CSN, CLASS, SO, ALTFLAG, ALTANN, ALTCLASS and ALTSO) will have empty value (e.g. ENST=.).

**9.2 SNP-based annotation**

If option flag @dbsnp is set in the configuration file, for each base substitution CAVA will search the dbSNP database file to find registered SNPs. If a variant is identified as a known SNP, the annotation flag 'DBSNP' will output its dbSNP identifier (e.g. DBSNP=rs206437).


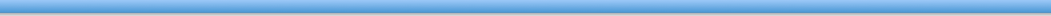


**10 THE CLASS ONTOLOGY**

As discussed in Section 9.1, variant calls overlapping with transcripts can be annotated with the 'CLASS' flag which refers to a simple ontology describing different types of variants. This ontology is described here in detail.

The values of the CLASS flag are described in the table below:

| **CLASS** | **Description** |
| --- | --- |
| SG | Stop-gain variant caused by base substitution. |
| ESS | Any variant that alters essential splice-site base (+1, +2, -1, -2). |
| SS5 | Any variant that alters the +5 splice-site base but not an ESS base. |
| SS | Any variant that alters splice-site base within the first @ssrange intronic bases flanking exon (i.e. +@ssrange to -@ssrange) but not an ESS or SS5 base. |
| EE | Variant that alters the first or last 3 bases of an exon (i.e. the *e*xon *e*nd), but not the frame of the coding sequence. |
| FS | Frameshifting insertion and/or deletion. It alters length and frame of coding sequence. |
| IM | Variant that alters initiating methionine start codon. |
| SL | Variant that causes a stop-loss (i.e. the stop codon is altered). |
| IF | Inframe insertion and/or deletion. It alters length but not frame of coding sequence. |
| NSY | Nonsynonymous variant. It alters amino acid(s) but not coding sequence length. |
| SY | Synonymous variant. It does not alter amino acid or coding sequence length. |
| INT | Any variant in an intron that does not alter splice-site bases. |
| 5PU | Any variant in 5’ untranslated region |
| 3PU | Any variant in 5’ untranslated region |

**Notes:**

**-** A variant can only have one CAVA class. If a variant could potentially be included in more than one class the first class in the list is assigned. For example, a frameshifting deletion that alters the start codon would be CAVA class FS (not IM).

- Nonsynonymous is also known as missense. Stop-gain is also known as nonsense.

- NSY and SY variants include complex variants that delete some bases from the coding sequence and insert different bases of an equal number. As a result, the length of coding sequence is not altered, therefore these variants are not the IF or FS classes.

- Indel variants that affect an exon-intron boundary are always classified as ESS variants.

- The @ssrange option in the configuration file can be used to define the size of the splice site region: i.e. the number of bases into the intron used as splice site in CLASS annotation. The parameter affects SS/INT boundary. The default value of @ssrange is 8 in order to make CLASS and SO ontologies comparable.

- Duplications overlapping the boundary of the 3’ splice site region of an intron are classified as INT as they do not alter splice site region: e.g. c.10-12dupC or c.10-13_10-11dupTGC (if @ssrange=12).

**The values of the CLASS flag have corresponding SO terms, with some noted exceptions, presented in the table below:**

| **CLASS** | **SO term** | **Exception** |
| --- | --- | --- |
| SG | stop_gained |  |
| ESS | splice_acceptor_variant, splice_donor_variant | The SO term is provided as appropriate for variants altering splice acceptor and splice donor sites, however the same CLASS value is returned for both types of variants. |
| SS5 | splice_donor_5th_base_variant |  |
| SS | intron_variant\|splice_region_variant | The SO term is always provided up to 8 bases into the intron to match the SO definition, however the CLASS value is provided according to @ssrange, so this will not be a one-to-one mapping when @ssrange value is not 8. |
| EE | splice_region_variant\|inframe_deletion, splice_region_variant\|inframe_insertion,  splice_region_variant\|synonymous_variant,  splice_region_variant\|missense_variant | The SO term is provided as appropriate for inframe deletions and insertions and base substitutions which affect the first and last three bases of the exon, however the same CLASS value is returned for all of these types of variants. |
| FS | frameshift_variant, splice_region_variant\|frameshift_variant | The SO term is provided as appropriate for frameshifting indels which do and do not affect the first and last three bases of the exon, however the same CLASS value is returned for both types of variants. |
| IM | initiator_codon_variant |  |
| SL | stop_lost |  |
| IF | inframe_deletion, inframe_insertion | The SO term is provided as appropriate for inframe deletions and insertions, however the same CLASS value is returned for both types of variants. |
| NSY | missense_variant |  |
| SY | synonymous_variant |  |
| INT | intron_variant | The SO term is always provided beyond 8 bases into the intron to match the SO definition, however the CLASS value is provided according to @ssrange, so this will not be a one-to-one mapping when @ssrange is not 8. |
| 5PU | 5_prime_UTR_variant |  |
| 3PU | 3_prime_UTR_variant |  |


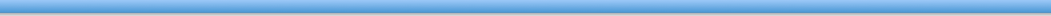


**11 OUTPUT**

Variant annotations discussed in Section 9 can be outputted by CAVA in two different file formats; VCF and CSV. The output format is specified in the configuration file by option flag @outputformat. The structure of output files are described in this section.

**11.1 Output in VCF format**

If VCF output format is used, variant call annotations are added to the INFO field of the VCF file. Following the VCF specification, annotation flags are separated by semicolons in the INFO field. For example:

TYPE=SUBSTITUTION;ENST=ENST00000379410;GENE=PLEKHN1;TRINFO=+/8.6kb/16/2.4kb;LOC=3UTR;CSN=c.*483A>G;CLASS=3PU;ALTFLAG=None;DBSNP=rs668558

If the input is a VCF file, the variant annotation flags are appended to the original INFO field values of each variant. The ID, CHROM, POS, REF and ALT fields in the output VCF file will be the same as the ID, chromosome, position, reference and alternative allele values in the input file; i.e. even if indels are left or right aligned in some transcript, the original genomic coordinates and alleles are given in the output. The QUAL and FILTER field values are also copied from the input file. Furthermore, if the input VCF file contains the FORMAT column and sample-specific genotype calls, these are also present in the output file.

**11.1.1 Multiallelic VCF records**

Multiallelic variant calls represented in a single VCF record in the input are also outputted in the same VCF record in the output. Each alternative allele has different TYPE, LOC, CSN, CLASS, SO, ALTFLAG, ALTANN, ALTCLASS, ALTSO and DBSNP flag values. If reported in a single VCF record, the multiple annotations corresponding to the different alternative alleles are comma-separated in the TYPE, LOC, CSN, CLASS, SO, ALTFLAG, ALTANN, ALTCLASS, ALTSO and DBSNP flags. For example, for the following triallelic VCF record describing a 1-base deletion and a 1-base insertion

#CHROM POS ID REF ALT …

8 3443799 . GA G,GAA …

the TYPE and CSN annotation flags are given as follows:

TYPE=Deletion,Insertion;

CSN=c.1098-18delT,c.1098-18dupT;

**11.1.2 Variants overlapping with multiple transcripts**

Variants that overlap with multiple transcripts have different ENST, GENE, TRINFO, LOC, CSN, CLASS, SO, ALTFLAG, ALTANN, ALTCLASS and ALTSO annotation flag values corresponding to the different transcripts. In this case, the multiple annotations values are colon-separated in these annotation flags. For instance, the following biallelic substitution overlaps with two transcripts in the whole exome database; ENST00000438763 and ENST00000452392.

#CHROM POS ID REF ALT …

6 32784783 . C T …

The ENST, CSN and CLASS annotations corresponding to the two transcripts are given as follows:

ENST=ENST00000438763:ENST00000452392;

CSN=c.-55G>A:c.1933-54G>A;

CLASS=5PU:INT;

**11.1.3 Multiallelic calls overlapping with multiple transcripts**

If a VCF record describes a multiallelic variant call that overlaps with multiple transcripts, the two rules above are combined: annotation values referring to different transcripts are colon-separated and values corresponding to different alternative alleles are comma-separated. For example, the following triallelic substitution overlaps with two transcripts in the whole exome set; ENST00000325203 and ENST00000344683.

#CHROM POS ID REF ALT …

8 6389889 . C G,A …

The GENE, LOC and CSN annotation flags are given as follows:

GENE=ANGPT2:MCPH1,ANGPT2:MCPH1;

LOC=Ex2:In12/13,Ex2:In12/13;

CSN=c.408G>C_p.=:c.2214+32439C>G,c.408G>T_p.=:c.2214+32439C>A

Note that both alternative alleles in both genes (ANGPT2 and MCPH1) cause synonymous and intronic changes, respectively.

**11.2 Output in CSV format**

If CSV output format is used, variant call annotations are written to a TAB-delimited TXT file that by default contains the following 17 columns:

1. ID (i.e. Variant call ID taken from the input file)

2. CHROM (i.e. chromosome of variant)

3. POS (i.e. genomic position of variant)

4. REF (i.e. reference allele of variant)

5. ALT (i.e. alternative allele of variant)

6. QUAL (i.e. QUAL value in the input VCF record)

7, FILTER (i.e. FILTER value in the input VCF record)

8. TYPE (i.e. value of TYPE annotation flag)

9. ENST (i.e. value of ENST annotation flag)

10. GENE (i.e. value of GENE annotation flag)

11. TRINFO (i.e. value of TRINFO annotation flag)

12. LOC (i.e. value of LOC annotation flag)

13. CSN (i.e. value of CSN annotation flag)

14. CLASS (i.e. value of CLASS annotation flag)

15. SO (i.e. value of SO annotation flag)

16. IMPACT (i.e. value of IMPACT annotation flag)

17. ALTFLAG (i.e. value of ALTFLAG annotation flag)

18. ALTCLASS (i.e. value of ALTCLASS annotation flag)

19. ALTSO (i.e. value of ALTSO annotation flag)

20. DBSNP (i.e. value of DBSNP annotation flag)

**11.2.1 Multiallelic calls and/or multiple transcripts**

Every line of the output CSV file represents a single variant call. Unlike in the VCF format, annotation information for multiallelic variant calls are split to multiple lines. If a variant call overlaps with multiple transcripts, the information is also split into multiple records. Two examples are shown below.

For the single VCF record in Section 11.1.1, two lines will be added to the output CSV file:

. 8 3443799 GA G 42 PASS Deletion ENST00000537824 CSMD1 …

. 8 3443799 GA GAA 42 PASS Insertion ENST00000537824 CSMD1 …

Another example is the single VCF record in Section 11.1.3 which will be represented by four lines in the output CSV file:

. 8 6389889 C G 200 PASS SUBSTITUTION ENST00000325203 ANGPT2 …

. 8 6389889 C G 200 PASS SUBSTITUTION ENST00000344683 MCPH1 …

. 8 6389889 C A 200 PASS SUBSTITUTION ENST00000325203 ANGPT2 …

. 8 6389889 C A 200 PASS SUBSTITUTION ENST00000344683 MCPH1 …


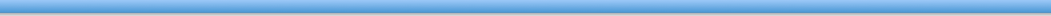


**12 VARIANT CALL FILTERING**

CAVA offers a number of different options to filter the input variant set so that only the selected subset of calls are annotated and written to the output file.

**12.1 Filtering by variant type**

Specified in the configuration file by flag @type, this option can be used to select only a particular variant type: substitutions, insertions, deletions, complex or all indels. Alternatively, setting the value to 'ALL' makes CAVA annotate and output every variant calls regardless of their type.

**12.2 Filtering by VCF filter**

CAVA can filter out variant call records based on the FILTER field of the input VCF file. If this option is switched on in the configuration file by the boolean flag @filter, CAVA will only annotate VCF records that have a PASS filter value.

**12.3 Filtering by BED file**

CAVA can apply a filter on variant calls based on a BED file describing a set of genomic regions of interest. If this option is used, the program will only annotate and output variants which overlap with any of the genomic regions specified in the BED file. The BED file should use 0-based genomic coordinates. It must be compressed by bgzip and indexed by Tabix. Both the bgzipped BED file and the index file (e.g. *panel.bed.gz* and *panel.bed.gz.tbi*) should be available in the same directory. The name of the BED file should be given in the configuration file at option flag @target.

**12.4 Filtering by gene list**

Another option CAVA offers for filtering is restricting variant annotations to a subset of genes of interest. In this case, only variant calls overlapping with particular genes are annotated and outputted. The list of gene (HGNC) symbols of interest should be given in a simple txt file (see an example below). The name of the txt file must be specified in the configuration file by option flag @genelist.

Example gene list txt file:

BRCA1

BRCA2

FANCD2

…

**12.5 Filtering by transcript list**

Similarly to filtering by gene list, CAVA can restrict variant annotations to a subset of transcripts of interest. In this case, only variant calls overlapping with particular transcripts are annotated and outputted. The list of Ensembl transcript IDs (ENST) should be given in a simple txt file (see an example below). The name of the txt file must be specified in the configuration file by option flag @transcriptlist.

Example transcript list txt file:

ENST00000380152

ENST00000358533

ENST00000338591

…

**12.6 Filtering by SNP list**

Finally, CAVA can filter variant annotations based on a list of selected SNPs. In this case, only base substitutions annotated with particular dbSNP identifiers are outputted. The list of dbSNP IDs should be given in a simple txt file (see an example below). The name of the txt file must be specified in the configuration file by option flag @snplist.

Example SNP list txt file:

rs4104967

rs206437

rs149472673

…

Note that if both @genelist and @transcriptlist are specified, in order to be outputted, variants have to satisfy both requirements: i.e. both their ENST and GENE annotations should be in the @transcriptlist and @genelist files, respectively. If @snplist is set, variants with DNSNP annotation found in the @snplist file are outputted regardless of whether their ENST and/or GENE annotations are listed in the @transcriptlist and @genelist files.

**12.7 Filtering non-annotated calls**

Some variants may neither overlap with any of the transcripts in the local Ensembl transcript dataset nor correspond to any SNPs in the local dbSNP dataset. These non-annotated variants get only one non-empty annotation flag; the TYPE flag. CAVA offers an option to filter out these non-annotated calls. If the option is switched on in the configuration file by option flag @nonannot, only variant calls with either non-empty ENST or DBSNP annotation flags are outputted.


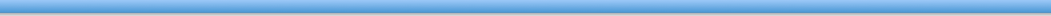


**13 THE DBPREP TOOL**

As discussed above, CAVA relies on local database files to perform transcript-based or SNP-based variant annotations. Both the Ensembl transcripts and dbSNP databases used by CAVA have specific formats and are compressed and indexed with Tabix. A simple tool, dbprep, is provided to generate the correct database files given a user-specified set of Ensembl or dbSNP identifiers.

**13.1 Creating transcript database file with dbprep**

This section describes how to generate the transcript database file given a list of Ensembl identifiers. The list of ENST IDs should be given in a text file. For example, let the file *transcripts.txt* contain:

ENST00000398334

ENST00000263121

ENST00000314074

...

dbprep will download the Ensembl trasncript database via FTP and retrieve relevant information about these transcripts. To generate the transcript database file, one can use the following command:

**python dbprep.py -i transcripts.txt –e 65 -o output**

Option -i specifies the name of input file containing the transcript IDs of interest. Note that if no input file is given, dbprep will retrieve all transcripts from the Ensembl database. Option -o defines the name of output file to be created by dbprep. Finally, option -e specifies the version of the Ensembl database. In the above example, Ensembl release 65 is used.

By default, dbprep downloads the transcript database created based on the human reference genome build GRCh37. It is possible to use a different reference genome build specified by the -g command line flag. (Note that genome build GRCh38 can only be used with Ensembl release 76 or later.) For example:

**python dbprep.py -i transcripts.txt –e 78 –g GRCh38 -o output**

Running the above example, dbprep will create an output file named *output.gz*, which is already the compressed and Tabix-indexed database file (an index file *output.gz.tbi* is also created). Running CAVA, one can refer to this database file in the configuration file by option flag @ensembl (see Section 4):

@ensembl = output.gz

**13.2 Creating dbSNP database file with dbprep**

dbprep can also be used to generate the correct dbSNP database file required by CAVA given a list of dbSNP identifiers. The list of dbSNP IDs should be given in a text file. For example, let the file *SNPs.txt* contain:

rs113145990

rs112294815

rs77918077

...

dbprep also requires the compressed VCF file (*00-All.vcf.gz*) released by NCBI containing all data about the entire set of SNPs in the dbSNP database. This file can be downloaded from the official FTP site of dbSNP:

ftp://ftp.ncbi.nih.gov/snp/organisms/human_9606/VCF/

In order to generate the dbSNP database file used by CAVA, given for instance dbSNP release 137, one can run the following command:

**python dbprep.py -i SNPs.txt -s 137 -d 00-All.vcf.gz -o output**

where option -i specifies the name of the input file containing the dbSNP IDs of interest, option -s specifies the version of the dbSNP release, option -d refers to the name of the vcf.gz file downloaded from the dbSNP FTP site and -o defines the name of the output file.

Running this command, dbprep will create an output file named *output.gz*, which is already the compressed and Tabix-indexed database file (an index file *output.gz.tbi* is also created). Running CAVA, one can refer to this database file in the configuration file by option flag @dbsnp (see Section 4):

@dbsnp = output.gz


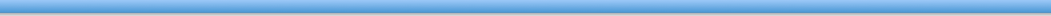


**14 DEFAULT TRANSCRIPT AND DBSNP DATABASE**

Although the dbprep tool gives the user flexibility to create any transcript and SNP database files, default transcript and SNP databases can also be downloaded from the project's webpage. The default transcript databases available include a whole human exome transcript set and a SNP set based on version 138 of the dbSNP database.

**14.1 Whole exome transcript database file**

The default *exome_65_GRCh37.gz* (and *exome_65_GRCh37.gz.tbi*) file contains 19650 transcripts from Ensembl release 65 representing the whole exome (the procedure of selecting these transcripts is described in detail in the CAVA publication and the list of Ensembl transcript identifiers can be downloaded from the CAVA website). The size of the database file is 1.9 MB.

As described above, the transcript database can be specified in the configuration file as

@ensembl = exome_65_GRCh37.gz

**14.2 dbSNP138 database file**

The default *dbSNP138.gz* (and *dbSNP138.gz.tbi*) file contains all SNP records from the dbSNP database release 138. A total number of 51989284 SNPs are included. The size of the file is 421 MB.

As described above, the SNP database can be specified in the configuration file as

@dbsnp = dbSNP138.gz


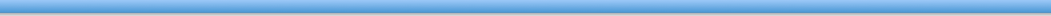


**15 RUN TIMES**

Running on a 2.9 GHz machine, CAVA can annotate 1.5+ million VCF records in one hour (i.e. 25000+ records per minute) using the default whole exome transcript dataset and dbSNP138 dataset.


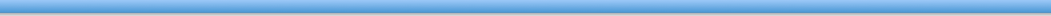


**16 LOG FILE**

If switched on in the configuration file by option flag @log, CAVA creates a log file during the annotation process, writing out status information and possible error messages. The name of the log file is created from the output file prefix defined at the command line (-o) to which the '.log' file extension is added.


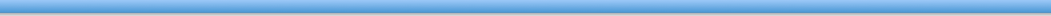


**17 EXAMPLES**

Three examples are presented in this final section explaining the configuration file, input file(s) and output file for each. Example 1 and 2 describe the scenario of annotating high quality variants overlapping gene transcripts from exome data, while Example 3 illustrates annotation of variants from a targeted panel.

**17.1 Example 1**

**17.1.1 Configuration file**

In the first example, the configuration file (*config.txt*) is as follows (see explanation below):

@inputformat = VCF

@outputformat = VCF

@reference = hg19.fa

@ensembl = exome_65_GRCh37.gz

@dbsnp = .

@nonannot = TRUE

@filter = FALSE

@type = ALL

@target = .

@genelist = .

@transcriptlist = .

@snplist = .

@logfile = FALSE

@givealt = FALSE

@ontology = CLASS

Both the input and output formats are set to VCF by option flags @inputformat and @outputformat, respectively. The reference genome (*hg19.fa*) located in the current directory is specified by option flag @reference. Transcript-based annotation is switched on (based on the default whole exome transcript database specified by option flag @ensembl). On the other hand, SNP-based annotation is switched off as @dbsnp is empty. Since option flag @nonannot is set to True, even variants that do not overlap with any transcripts are outputted. No filtering will be performed based on the FILTER field of the input VCF file as @filter is set to False. CAVA will output all types of variants (@type=ALL). Since @target, @genelist, @transcriptlist and @snplist are all set empty, no further variant filtering is carried out. Only the CLASS ontology will be reported for variants (@ontology=CLASS). Alternative most 5’ sequence and CLASS annotations are not reported, only the ALTFLAG flag is outputted (@givealt=FALSE). Finally, as option flag @log is set to False, no log file is created.

**17.1.2 Input file**

The input file (*input.vcf*) is in VCF format containing 3 records, one of which represents a triallelic variant call:

#CHROM POS ID REF ALT QUAL FILTER INFO

9 137735017 1 T TAGGG 200 PASS ANYFLAG=1

13 32915330 2 GTGGGTAAGT G 926 PASS ANYFLAG=4

3 197566254 3 T C,A 200 PASS ANYFLAG=2

Note that these input VCF records already have information in their INFO field (“ANYFLAG”).

**17.1.3 Running CAVA**

CAVA is run by the following command; command line argument -c specifying the configuration file name, argument -i specifying the input file name and -o specifying the prefix of output file name:

**python path/to/cava/cava.py -c config.txt -i input.vcf -o output**

**17.1.4 Output file**

The output file (*output.vcf*) created by CAVA follows VCF format and it contains the same CHROM, POS, ID, REF, ALT, QUAL and FILTER values as the input VCF file. However, the INFO fields of the three VCF records contain additional information about the results of variant annotation.

**INFO field of first VCF record:**

ANYFLAG=1;TYPE=Insertion;ENST=ENST00000371817;GENE=COL5A1;TRINFO=+/203.1kb/66/8.5kb;LOC=3UTR;CSN=c.*870_*873dupGGGA;CLASS=3PU;IMPACT=3;ALTFLAG=AnnNotClass

**Explanation:** According to the INFO field, the first VCF record describes a 4-base insertion (duplication) in the gene COL5A1 affecting the 3’ untranslated region of the ENST00000371817 transcript. The transcript is forward-stranded, has a length of 203.1 kb including 66 exons which together make up 8.5 kb of coding sequence. The CSN annotation of the variant is ‘c.*870_*873dupGGGA’. The insertion is classified as a 3PU (3’ UTR) variant with an impact level of 3, and has an alternative indel representation.

**INFO field of second VCF record:**

ANYFLAG=4;TYPE=Deletion;ENST=ENST00000380152;GENE=BRCA2;TRINFO=+/83.7kb/27/10.9kb;LOC=Ex11-In11/12;CSN=c.6839_6841+6del9;CLASS=ESS;IMPACT=1; ALTFLAG=None

**Explanation:** According to the INFO field, the second VCF record describes a 9-base deletion in the gene BRCA2 overlapping the boundary of Exon 11 and Intron 11/12 in the ENST00000380152 transcript. The transcript is forward-stranded, has a length of 83.7 kb including 27 exons which together make up 10.9 kb of coding sequence. The CSN annotation of the variant is ‘c.6839_6841+6del9’. The deletion is classified as an ESS (essential splice site) variant, because the +1 and +2 bases are deleted with an impact level of 1, and has only one indel representation.

**INFO field of third VCF record:**

ANYFLAG=2;TYPE=SUBSTITUTION, SUBSTITUTION;ENST=ENST00000334859,ENST00000334859;GENE=LRCH3,LRCH3;TRINFO=+/80.3kb/19/2.3kb,+/80.3kb/19/2.3kb;LOC=Ex10,Ex10;CSN=c.1314T>C_p.=,c.1314T>A_p.Tyr438X;CLASS=SY,SG;IMPACT=3,1;ALTFLAG=None,None

**Explanation:** According to the INFO field, the third VCF record describes a triallelic variant with both alternative alleles representing a base substitution. The substitutions are located within the gene LRCH3, in Exon 10 of the ENST00000334859 transcript. The transcript is forward-stranded, has a length of 80.3 kb including 19 exons which together make up 2.3 kb of coding sequence. The two alternative alleles are flagged with different CSN and CLASS annotations. The CSN annotation of the first base substitution is ‘c.1314T>C_p.=’ and it is classified as a SY (synonymous) variant with an impact level of 3. By contrast, the CSN annotation of the second substitution is ‘c.1314T>A_p.Tyr438X’ and it is classified as an SG (stop-gain) variant as amino acid Tyr438 changes into a stop codon and has impact level of 1).

**17.2 Example 2**

**17.2.1 Configuration file**

In the second example, the configuration file (*config.txt*) is as follows (see explanation below):

@inputformat = VCF

@outputformat = CSV

@reference = hg19.fa

@ensembl = exome_65_GRCh37.gz

@dbsnp = dbSNP138.gz

@nonannot = FALSE

@filter = TRUE

@type = DELETION

@target = .

@genelist = genelist.txt

@transcriptlist = .

@snplist = .

@givealt = FALSE

@logfile = TRUE

@ontology = CLASS

The input format is set to VCF, the output format is set to CSV by option flags @inputformat and @outputformat, respectively. The reference genome (*hg19.fa*) is specified by option flag @reference. Both transcript-based and SNP-based annotations are performed (using the default whole exome transcript database and the dbSNP138 database specified by option flags @ensembl and @dbsnp, respectively). Unlike in the previous example, non-annotated variants are not written to the output file (@nonannot=FALSE). In addition, records with no PASS value in their VCF FILTER field are also filtered out (@filter=TRUE). As set by option flag @type, only deletions are considered for annotation. Furthermore, a text file containing a list of gene identifiers is provided (specified by option flag @genelist). Only variants that overlap with at least one gene found on this list are outputted. Only the CLASS ontology will be reported for variants (@ontology=CLASS). Alternative most 5’ sequence and CLASS annotations are not reported, only the ALTFLAG flag is outputted (@givealt=FALSE). Finally, since option flag @log is set to TRUE, a log file is being created during variant annotation.

**17.2.2 Input file**

The input file (*input.vcf*) is in VCF format containing 7 records; 6 deletions and 1 base substitution:

#CHROM POS ID REF ALT QUAL FILTER INFO

1 152192825 1 C T 200 PASS .

1 152195728 2 AT A 200 PASS .

5 176070830 3 GC G 78 PASS .

5 176071205 4 AG A 106 PASS .

7 25266569 5 TTAA T 200 PASS .

17 15341736 6 CAG C 54 SomeFilter .

17 15670733 7 CA C 20 PASS .

**17.2.3 Gene list file**

Furthermore, let the gene list file (*genelist.txt*) contain the following four gene symbols:

HRNR

EIF4E1B

BRCA1

BRCA2

**17.2.4 Running CAVA**

The same as in Section 17.1.3.

**17.2.5 Output file**

The output file (*output.txt*) created by CAVA follows the TAB-separated CSV format:

| ID | CHROM | POS | REF | ALT | QUAL | FILTER | TYPE | ENST | GENE | TRINFO | LOC | CSN | CLASS | IMPACT | ALTFLAG | DBSNP |
| --- | --- | --- | --- | --- | --- | --- | --- | --- | --- | --- | --- | --- | --- | --- | --- | --- |
| 2 | 1 | 152195728 | AT | A | 200 | PASS | Deletion | ENST00000368801 | HRNR | -/12.1kb/3/9.6kb | Ex2 | c.1delA | FS | 1 | AnnAndClass | . |
| 3 | 5 | 176070830 | GC | G | 78 | PASS | Deletion | ENST00000318682 | EIF4E1B | +/16.0kb/9/2.0kb | In5/6 | c.296+97delC | INT | 4 | AnnNotClass | . |
| 4 | 5 | 176071205 | AG | A | 106 | PASS | Deletion | ENST00000318682 | EIF4E1B | +/16.0kb/9/2.0kb | In5/6 | c.297-170delG | INT | 4 | AnnNotClass | . |

Out of the seven input VCF records, only three variant calls are outputted. Record 1 is not included in the output because it is a substitution and only deletions are annotated in this example. Record 5 is filtered out because this deletion affects the gene NPVF which is not present on the list in *genelist.txt*. Record 6 is excluded because it has a non-PASS FILTER value in the input VCF file. Finally, record 7 does not overlap with any Ensembl transcript so it is filtered out as a non-annotated variant.

The first variant in the output file (record 2) describes a single base deletion within the gene HRNR, in Exon 2 of the ENST00000368801 transcript. The transcript is reverse-stranded, has a length of 12.1 kb including 3 exons which together make up 9.6 kb of coding sequence. The CSN annotation of the variant is ‘c.1delA’. The deletion is classified as an FS (frame-shift) variant and has an alternative indel representation which affects its class. The other two outputted variants are also single base deletions, both within the gene EIF4E1B, in Intron 5/6 of the ENST00000318682 transcript. The transcript is forward-stranded, has a length of 16.0 kb including 9 exons which together make up 2.0 kb of coding sequence. The CSN annotations of the two deletions are ‘c.296+97delC’ and ‘c.297-170delG’, respectively. Both are classified as INT (intronic) variants and have alternative indel representations.

**17.2.6 Log file**

The following log file (*output.log*) is written during variant annotation:

2014-04-03 12:52:47,850 INFO: CAVA v1.0.0 started.

2014-04-03 12:52:47,850 INFO: Configuration file - config.txt

2014-04-03 12:52:47,850 INFO: Input file (VCF) - input.vcf

2014-04-03 12:52:47,850 INFO: Output file (CSV) - output.txt

2014-04-03 12:52:47,851 INFO: Connected to reference genome.

2014-04-03 12:52:47,851 INFO: Connected to Ensembl database.

2014-04-03 12:52:47,851 INFO: Connected to dbSNP database.

2014-04-03 12:52:47,851 INFO: Gene list loaded.

2014-04-03 12:52:47,851 INFO: 7 records to be annotated.

2014-04-03 12:52:47,851 INFO: Variant annotation started.

2014-04-03 12:52:47,852 INFO: 10% of records annotated.

2014-04-03 12:52:47,864 INFO: 20% of records annotated.

2014-04-03 12:52:47,866 INFO: 30% of records annotated.

2014-04-03 12:52:47,868 INFO: 40% of records annotated.

2014-04-03 12:52:47,872 INFO: 50% of records annotated.

2014-04-03 12:52:47,873 INFO: 60% of records annotated.

2014-04-03 12:52:47,874 INFO: 100% of records annotated.

2014-04-03 12:52:47,874 INFO: Output file = 0.4 Kbyte

2014-04-03 12:52:47,874 INFO: CAVA successfully finished.

**17.3 Example 3**

**17.3.1 Database preparation**

The third example illustrates how one can generate and use custom transcript and SNP databases instead of using the default sets. Let the file *transcripts.txt* contain the following Ensembl transcript identifiers of interest:

ENST00000327337

ENST00000358821

ENST00000369902

The dbprep tool is used to generate the corresponding transcript database (based on Ensembl release 65):

**python path/to/cava/dbprep.py -i transcripts.txt –e 65 -o custom_transcripts**

Similarly, let the file *snps.txt* contain the following dbSNP identifiers of interest:

rs11147489

rs4263028

rs111588517

The corresponding SNP database (based on dbSNP version 138) is generated by

**python path/to/cava/dbprep.py -i snps.txt -s 138 -d 00-All.vcf.gz**

**-o custom_SNPs**

**17.3.2 Configuration file**

The configuration file (*config.txt*) is as follows (see explanation below):

@inputformat = TXT

@outputformat = VCF

@reference = hg19.fa

@ensembl = custom_transcripts.gz

@dbsnp = custom_SNPs.gz

@nonannot = FALSE

@filter = FALSE

@type = SNP

@target = .

@genelist =

@transcriptlist = .

@snplist = .

@logfile = FALSE

@givealt = FALSE

@ontology = CLASS

The input format is set to TXT, the output format is set to VCF by option flags @inputformat and @outputformat, respectively. Both transcript-based and SNP-based annotations are performed, using the custom transcript and dbSNP databases generated above (*custom_transcripts.gz* and *custom_SNPs.gz*). As in the previous example, non-annotated variants are not written to output (@nonannot=FALSE). Finally, only base substitutions are annotated and outputted (@type=SUBSTITUTION).

**17.3.3 Input file**

The input file (*input.txt*) is in TXT format (see Section 5.2) containing five records, one of which describes a triallelic call.

#ID CHROM POS REF ALT

1 12 50745863 C A,G

2 12 50759433 AAATG A

3 13 32906980 A G

4 13 32929478 C T

5 18 72228124 A G

**17.3.4 Running CAVA**

The extension of input file is changed to TXT:

**python path/to/cava/cava.py -c config.txt -i input.txt -o output**

**17.3.5 Output file**

The output file (*output.vcf*) created by CAVA follows VCF format and the results of variant annotation are reported in the INFO field. Out of the 5 input records only 3 are present in the output. Record 2 is filtered out as it is not a substitution. Record 3 is excluded because, although it is a substitution affecting BRCA2, this gene is not included in the custom transcript database, therefore the variant is considered non-annotated. Record 4 is written to the output because its dbSNP identifier is included in the custom SNP database. As records 1 and 5 describe substitutions in the FAM186A and CNDP1 genes which are included in the custom transcript database, these variants are written to the output file.

As shown by the INFO field of record 1, this triallelic substitution is located within the gene FAM186A, in Exon 4 of the ENST00000327337 transcript. The transcript is reverse-stranded, has a length of 69.2 kb including 8 exons which together make up 7.1 kb of coding sequence. The CSN annotations for the different alternative alleles are ‘c.4752G>T_p.=’ and ‘c.4752G>C_p.=’, respectively. Both are classified as SY (synonymous) variants.

The INFO field of record 2 reports that this substitution does not overlap with any transcripts in the custom database but has a dbSNP identifier (rs11147489).

Finally, as shown by the INFO field of record 5, this substitution is located within the gene CNDP1, in Exon 4 of the ENST00000358821 transcript. The transcript is forward-stranded, has a length of 50.6 kb including 12 exons which together make up 2.2 kb of coding sequence. The CSN annotation is ‘c.337A>G_p.Ile113Val’ and the variant is classified as NSY (non-synonymous) variant. It also has a dbSNP identifier reported (rs4263028).


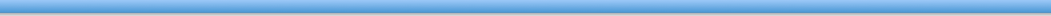


**18 CONTACT**

Please send any bug reports, comments or feature requests to:

[cava-user-group@googlegroups.com](mailto:cava-user-group@googlegroups.com)
